# Supplementary material for: Are carbon emissions trading and green financial instruments synergistic? -Comprehensive quantitative research based on content analysis
Source: PLoS One. 2024 Mar 7;19(3):e0298601. doi: 10.1371/journal.pone.0298601 (PMC10919601; doi:10.1371/journal.pone.0298601)
Supplement: S1 Data — (ZIP) [file pone.0298601.s001.zip › Data/地级市Z_F工作报告.zip!/║┌┴·╜¡/╥┴┤║╩╨/└┤╫╘╣1⁄2╓┌║┼í╛┬φ┐╦╩2╛▌═°í┐_2016.docx]

各位代表：

现在，我代表市政府向大会报告工作，请予审议，并请市政协委员和其他列席会议的同志提出意见。

一、“十二五”时期及2015年工作回顾

“十二五”时期是伊春发展史上极不寻常、极不平凡的五年。面对转型发展中的各种矛盾和困难，在市委的坚强领导下，在市人大、市政协的有力监督支持下，我们紧紧依靠全市人民，抢抓机遇，攻坚克难，圆满完成了“十二五”规划确定的目标任务，经济社会建设取得了重大进展。

——深入实施天保二期工程和《大小兴安岭林区生态保护与经济转型规划》，全面停止商业性采伐，森林生态系统得到休养生息。森林覆盖率由2010年的 83.9%提高到84.4%，林木总蓄积由2.56亿立方米提高到2.92亿立方米，首批跨入国家生态文明先行示范区行列。

——强力推进产业项目和园区建设，着力构建“3+X”生态主导型产业体系，经济增长动能由“钢木支撑”向“多极驱动”加速转换。连续启动实施两轮产业项目攻坚战，推动建设省市级以上重大产业项目96个，总投资223亿元。亚洲最大的鹿鸣钼矿建成投产。铁力、翠峦园区、嘉荫对俄跨境经济合作区等产业园区加速推进，承载和带动能力持续增强。光明家具实现二次崛起，成为全国实木家具网上销售的典范。森林食品产业“红蓝黑黄+林药+林畜”6条产业链渐成规模，汤旺河景区晋升国家5A级景区。“钢木经济”占GDP比重由2010年的18.9%降至4.4%，生态旅游和森林食品业占比分别上升5.7和5.2个百分点。

——稳步推进重点领域改革，持续开展以大招商为重点的对外开放，经济社会发展活力日益增强。以林业综合改革为统领，政企合一区公务员登记和森工企业内部经营机制改革取得实质性进展。投融资体制改革深入推进，融资渠道逐步拓宽。森林经营碳汇交易在伊春率先开市，成立中国林权交易所-伊春交易中心。行政审批流程不断简化，城市管理综合执法和社区扁平化管理改革走在全省前列。积极实施全方位对外开放战略，成功引进中铁、中建、华能等央企，以及汇源、佳龙等战略投资者，境外采伐规模进一步扩大，对外开放合作迈出新步伐。

——以建设国际化森林旅游名城为目标，全面推进重大基础设施建设，城市功能和品位同步提升。铁金、浩南公路建成通车，“三纵两横”路网全面升级。华能热电“双投”并网，新建垃圾和污水处理厂24个，“三供两治”能力全面增强。以“创文”和承办省运会、森博会为牵动，八大体育场馆和汇源国际会展中心、水上公园、沿河公园、步行街、比罗比詹广场等一批高品位公园广场建成投用，绿色伊春新形象全面展示。

——切实保障基本民生，全面增强公共服务能力，和谐平安伊春建设取得重大进展。组织实施了历史上最大规模的林区棚改，67万林区群众告别了居住近半个世纪、低矮破旧的棚户区，人均住房面积增长了近1倍。各级各类教育协调发展，高考实现十连升，市一中跨入全省名校行列。医疗卫生事业取得较大进步，市中心医院和省林业二院相继晋级“三甲”医院，基层医疗机构水平大幅提升。大力实施文化惠民工程，图书馆、艺术馆、博物馆免费向市民开放。大集体、五七工、小知青等历史遗留问题陆续解决，森工企业社会保险实现了应保尽保。林业职工年均工资由2010年的12684元提高到31524元，增长1.5倍，企业退休人员养老金翻了一番。在停伐木材收入“清零”、财政收入骤减的情况下，坚持民生投入只增不减，五年累计投入312亿元。“十二五”期间相继获得国家卫生城市、龙江首个全国社会管理综合治理最高荣誉“长安杯”、国家六星级慈善城市、全国幸福城市等诸多荣誉，城乡居民的获得感和幸福指数不断攀升!

各位代表，刚刚过去的2015年是极不寻常的一年，全市经济社会发展面临十分严峻的挑战，资源性、体制性、结构性矛盾更加突出，各种问题交替显现，压力超乎寻常。立足转型发展的特殊节点，我们牢牢把握省委对伊春提出的“在全省生态文明建设和转型发展中发挥带头作用”的定位，保持定力，积极作为，统筹推进稳增长、调结构、促改革、惠民生等各项工作，努力开创经济社会发展新局面。

——经济下行趋势得到有效遏制。突出针对性、实效性，制定了系列稳增长政策措施，积极帮助西钢等重点企业稳定生产、开拓市场，力促经济止滑企稳。全年地区生产总值完成248.2亿元，同比下降2.7%，降幅收窄6.7个百分点;固定资产投资完成101亿元，同比下降23.8%，降幅收窄18.8个百分点;公共财政预算收入完成14.2亿元，同比下降2.6%;社会消费品零售总额实现107.7亿元，同比增长9.9%。经济指标下滑的因素主要是：经济结构不优，短期内还无法适应全国“三期”叠加、过剩产能调整及全面停伐等宏观政策带来的深度影响，全市传统经济增长受到较大冲击;“十二五”前期棚改、基础设施建设、西钢技改等政策导入性投入短期内做大了基数，从统计上对增速回落产生了直接影响;新的替代产业规模还不够大，品牌影响还不够强，税收还未形成较大支撑能力，等等。

——产业升级步伐加快。森林生态旅游、森林食品、北药、绿色矿业四大产业板块加速构建。西岭、龙湾旅游度假区、森林冰雪玉温泉等重点旅游项目全面建设，锦绣山水公园晋升国家4A级景区，哈尔滨-铁力-伊春森林冰雪旅游被列入省级冬季主推精品线路，全年游客人次和旅游收入分别同比增长13.5%和 14.5%。森林食品6条产业链不断延伸，食用菌规模达5.1亿袋，森林猪饲养量达20万头，成功获批“国家级出口森林食品质量安全示范区”。北药种植和改培面积稳中有增，各类药材基地达97个。鹿鸣钼矿全面投产，与上海海派玉雕文化协会达成了北红玛瑙产业合作框架协议。成功举办了首届木制工艺品展览会，木艺家具传统产业优势进一步放大。深入实施“互联网+”战略，电商产业园投入运营，“淘宝·特色中国·伊春馆”等线上平台全面开通，淘宝大学电商培训基地落户伊春。坚持项目园区两手抓，全年实施重点产业项目23个，铁力园区累计入驻企业26户;翠峦园区建设“三年攻坚战”和“180招商计划”全面启动，园区内小微企业创业园被评为“国家级首批创业创新示范基地”。

——生态建设有效开展。全面巩固停伐成果，依法打击乱占林地、毁林开垦等行为，查处各类资源林政案件94起，恢复林地8.2公顷。加大森林培育力度，全年完成更新造林35万亩，中幼龄林抚育287万亩，实现了连续12年无重大森林火灾。湿地、野生动植物资源得到有效保护。顺利通过省级生态市创建考核验收，伊春国家公园体制试点被确定为全国首批9个试点之一。空气综合质量在全省地市中排名第一。

——改革开放稳步推进。认真贯彻中央6号文件精神，积极参与《黑龙江省国有重点林区改革方案》的编制工作，五营、新青、铁力、红星4个改革试点有序开展，森林资源和林业资产清产核资全面推进。大公国际信用评估集团、哈慈股份、华电兴安汇公司同步落户伊春，为推动资源与资本市场对接奠定了坚实基础。大力简政放权，共取消下放暂停事项150项，在全省率先推行了“四证合一”登记制度。举办了首届伊春国际森林马拉松赛、森林食品交易会等重大会展赛事活动，承办了第七届东北东部(12+2)区域合作圆桌会议，进一步促进了区域开放融合。

——城乡建设提质提效。全年建设保障性住房2.2万套，完成居民楼外墙节能改造172万平方米，改造老旧小区20个。龙湾大街续建、伊翠铁路改线等基础设施工程进展顺利。中心城净水厂项目开工建设，12座污水处理厂建成投用。山河大道、丁香园及各区局重点景观带绿化美化工程同步推进。全面启动实施了美丽乡村、林场所建设三年行动。顺利通过国家卫生城市复审，城乡环境进一步巩固提升。

——人民生活持续改善。坚持就业创业优先，全市城镇新增就业2.3万人。持续提高社会保障和公共服务水平，全面推开了城乡居民大病保险，启动了新型农村合作医疗及城镇居民基本医疗保险市级统筹，完成了11.6万厂办大集体人员身份认定工作，为森工企业在职职工月增资600元。有效推进铁力市、嘉荫县公立医院试点改革，建设医疗联合体30个。养老机构每千名老人拥有床位数提前达到省标，示范型、标准型社区服务中心占比位居全省前列。成功举办第十届市运会暨第二届全民健身运动会。全市义务教育标准化学校达标率95%，高考本科二表以上入段率达到61.8%，高出全省10.7个百分点。民生支出“量比齐增”，较上年增加15.8亿元，占公共预算支出的比重达到75%，创历史新高。在全面完成年初承诺的15件民生实事的基础上，还推进了“暖桥”、垃圾中转站、给水分户改造等一批民生工程建设。

各位代表!五年的持续攻坚，五年的阔步前行，开创了全市转型发展的良好局面，奠定了全面建成小康社会的坚实基础。在此，我代表市政府，向全市人民、驻伊部队指战员、武警官兵、公安干警，向各民主党派、人民团体，致以崇高的敬意!向关心支持伊春发展的中省直部门，及所有参与伊春建设的劳动者和各界人士，表示衷心的感谢!

各位代表!五年的工作实践，进一步深化了我们对林区经济社会发展规律的认识，提升了我们在复杂环境中推动科学发展的能力。我们深深体会到，伊春的转型发展，必须坚持党的领导，始终做到思路明晰、措施有力，确保中央和省市委决策部署落地生根;必须坚持生态为先，牢固树立“绿水青山就是金山银山”的发展理念，坚定不移走绿色化转型发展之路;必须坚持发展为上，把产业项目建设作为伊春加快转型发展的根本出路和核心任务，推动产业向中高端迈进;必须坚持创新为魂，以深化改革扩大开放为动力，向创新要活力，在更大空间谋划伊春、发展伊春;必须坚持民生为本，始终把人民对美好生活的向往，作为我们的奋斗目标;必须坚持担当尽责，以坚韧不拔、一往无前的劲头抓工作落实，切实做到善始善终、善做善成。

同时，我们也清醒地认识到，伊春转型攻坚还面临诸多矛盾和问题。主要是：产业结构仍处于调整培育期，经济增长动能不强;市场化程度不高、市场主体活力不足，发展环境仍需改善;偿债压力与民生投入之间的矛盾持续加大;面对经济发展新常态，一些政府公务人员缺乏担当意识，存在懒政怠政等现象，作风建设有待进一步加强。对此，我们必须从根本上把握，持之以恒地全力攻坚、一以贯之地全面改进!二、“十三五”时期的主要目标任务

根据市委十一届五次全会精神，市政府制定了《伊春市国民经济和社会发展第十三个五年规划纲要》，已提请大会审议。

“十三五”时期，全市经济社会发展的主要目标是：经济综合实力实现新提升，地区生产总值年均增长6%以上，比2010年翻一番;生态功能区建设实现新突破，完成天保工程二期任务目标，森林覆盖率稳定在85%左右，生态屏障作用更加稳固;经济转型实现新跨越，绿色生态产业得到快速发展，发展动能全面转换;国有林区改革取得决定性成果，资源性、体制性、结构性矛盾深度破解，林区经营管理体制基本理顺;城镇化建设质量整体提升，基础设施全面改善，城镇集聚能力和服务功能显著增强;人民生活水平和质量大幅提高，公共服务体系更加健全，就业更加充分，贫困人口全部脱贫。

实现上述总体目标，是市委赋予的重要责任，是全市人民的热切期待，是各级政府的使命之所在。我们深感肩上沉甸甸的压力，因为我们已清醒地看到，彻底保护住伊春这片大森林，老百姓的生存和发展问题不得到很好的解决不行;解决好百姓的生存和发展没有强大的经济基础不行;强大经济基础的形成，没有强大的产业体系支撑不行;强大产业体系的形成没有外部的重投资、大投资也不行。我们深知，伊春的发展，靠国家政策支持是必须的，但完全依赖国家政策过日子是不够的，也不会完全富裕起来，更无法完成省委赋予伊春“两个定位”的神圣使命。我们必须紧紧依靠市委的坚强领导，高举生态文明建设的大旗，坚定不移地走绿色化转型的道路，在全面落实好国家政策的同时，不断深化改革开放，不断推进创业创新，不断加快市场化步伐，大力发展林下经济，发展林区工业，努力探索符合伊春实际的转型发展路径。以踏石留印、抓铁有痕的务实态度，以夙夜在公、功成不必在我的精神境界，全力以赴，驰而不息地抓好各项工作的落实。突出把握五项重点任务：

一是加快推进产业结构战略性调整，努力实现内生驱动、特色多元的创新发展。坚持按照“做大一产、做优二产、做强三产”的思路，大力发展林下经济，大力推进产业发展。加快提升森林食品、北药、森林生态旅游、木业加工、绿色矿业五大产业的质量和效益，全面推动电子商务、会展物流、文化体育等现代服务业，有效促进经济结构优化升级，全面增强转型发展内生动力。

二是加快推进生态文明先行示范区建设，努力实现资源增长、生态优良的绿色发展。坚持按照主体生态功能区定位，以保护优先、自然恢复为主，严守生态保护红线，落实耕地保护制度，加强森林资源培育，放大国家公园体制试点效应，推进资源节约循环利用，全面筑牢生态安全屏障，守望我们的绿色家园。

三是加快推进改革开放，努力实现活力释放、动力增强的开放发展。坚持向机制创新和科技创新要转型发展动力，全面推进国有林区改革，全力探索资源资产化、资产资本化、资本证券化有效路径，加快民营经济发展，发挥市场在资源配置中的决定性作用。全面提升科技研发转化能力，增强科技对经济增长的支撑力。切实融入“龙江丝路带”，着力打造以对俄合作为重点的全方位开放新格局。

四是加快推进新型城镇化，努力实现城乡一体、互补融合的协调发展。顺应林业城市发展规律，统筹空间、规模、产业三大要素，调整优化城镇布局，引导人口合理集聚，加快提高城镇承载力和辐射力。以实现人的城镇化为核心，协调推进城乡重大基础和公共服务设施建设，持续开展文明城市、卫生城市、平安城市建设，加快形成良性互动、协调共进的城乡发展新局面。

五是加快推进民生改善，努力实现保障有力、富裕和谐的共享发展。坚持按照“普惠性、保基本、均等化、可持续”的原则，从解决人民最关心最直接最现实的利益问题入手，全力实施重点民生工程，持续加大就业社保、教育医疗、文化体育等社会事业投入，打赢脱贫攻坚战，让全市人民同步分享改革发展成果，生活得更加幸福、更有尊严。三、2016年主要工作安排

今年是“十三五”开局之年，根据市委部署，政府工作的总体要求是：全面贯彻落实党的十八大和十八届三中、四中、五中全会，中央经济工作会议，省委十一届六次全会和省委经济工作会议精神，按照“五位一体”总体布局和“四个全面”战略布局，牢固树立创新、协调、绿色、开放、共享的发展理念，积极适应经济发展新常态，紧紧抓住全面振兴东北等老工业基地机遇，创新实施“五大规划”发展战略，深入推进“龙江丝路带”建设，坚持稳中求进工作总基调，着力加强结构性改革，加快培育和发展五大支柱产业，保生态、促改革、稳增长、调结构、惠民生、防风险，精准发力，扎实起步，坚定不移走绿色化转型发展之路，努力实现“十三五”经济社会发展良好开局。

综合考虑全面建成小康社会的需要和现实可能，今年全市经济社会发展的预期目标是：地区生产总值增长6%，固定资产投资增长5%，规模以上工业增加值增长3%，公共预算收入增长5%，城乡居民收入增长与经济增长同步。

实现上述目标，要重点做好以下七项工作：

(一)持续加快产业结构调整。深入挖掘“供给侧”与“需求侧”潜力，以林下经济和林区工业为支撑，统筹推进五大支柱产业发展，带动经济结构优化升级。

大力发展森林食品业，突出市场开拓和品牌整合。森林食品是我们最有特色、最具资源优势的板块，必须努力克服产业层次低，规模小，缺乏品牌影响力，缺乏整体性营销等短板，主动出击，对接消费导向，抓住基地、加工、营销、品牌等环节，提升产业竞争力。全力支持汇源、中盟、黑尊、宝宇、日林、泓洋、北货郎、永旺等龙头项目达产达效。稳步扩大红松、蓝莓等坚果、浆果及有机大豆种植面积，稳定食用菌生产规模。着力推进以“两牛一猪”、森林鸡、森林鹅、寒地毛皮动物为重点的生态畜牧养殖加工基地和良种繁育基地建设。支持山庄经济发展，鼓励开展以互联网为载体的森林食品私人和集团定制，加速构建C2B商业模式。持续优化铁力市、嘉荫县农业种植结构，向优质、高效农业方向调整，扩大绿色水稻、甜玉米等有机作物种植面积，有效解决农业存在的种强销弱、量大链短、质优价不优等问题。推动农业由种得好向卖得好转变，以卖得好倒逼种得更好，提高农业的附加值，促进农民增收。支持汇源、升辉等物流建设，着手打造升辉绿色食品大市场，提升电商平台的规模和水平，鼓励微电商发展，市电商产业园力争晋升省级产业园，为企业“+互联网”创造便利，促进线上线下互动营销。深入实施商标战略，加强商标资源整合，集中打造并叫响“小兴安岭大森林”品牌。

大力发展北药业，突出药企引进和基地建设。北药是我们基础比较坚实、环境竞争优势明显的板块，必须努力克服种加脱节、布局分散、金融保险不配套等短板，走产业化发展路径，打造北药产业基地。按照战略性支柱产业发展定位，以做大增量为重点，种植上突出单品种、规模化，加工上突出龙头企业落地和培育，机制上突出企业+基地+农户等模式，重点推动国药集团红星平贝饮料、哈药四厂铁力中药材种植加工等项目落地，支持葵花、格润、天一等药企加快技改和精深加工项目建设。紧密对接药企需求，强化与大专院校、科研院所、医疗机构的合作，加强刺五加、苍术、返魂草等骨干品种标准化、规模化基地建设。抓好GAP、 GMP质量认证，叫响“林都北药”品牌。

大力发展森林生态旅游业，突出市场化经营和体制创新。森林生态旅游是我们整体性特征明显、生态性特征突出、外部性需求旺盛的板块，也是我们转型发展的重要牵动力量，必须努力克服体制机制不活、低水平重复建设、消费水平不高等短板，打造生态旅游新高地。深入推进国有景区经营模式创新，组建文旅集团，推进旅游产业上市融资。支持景域集团等社会资本参与景区开发和委托经营。积极创建“国家全域旅游示范区”，探索采取“景区联票”、“一卡通”等管理方式，支持铁力、桃山等南线精品线路打造，支持友好、五营、汤旺河、嘉荫等北线产品提升，支持美溪、金山屯等东线旅游拓展，支持伊春区“两山一园”产品开发及夜场做大。大力发展中医药养生、温泉度假、湿地旅游、精品民宿等新业态，推进友好汇鑫源旅游综合体、乳影岛温泉度假、新青户外运动谷、金山屯九峰山、嘉荫恐龙园扩建等项目建设。联动发展温泉、冰壶、滑雪、民俗项目以及室内水上项目，搅热冬季旅游。强化整体营销，加强与携程、同程等知名旅行社合作，拓展线上线下终端市场。提升旅游综合服务水平，建设游客到访中心。实施“旅游+”战略，着重推动文化、会议会展等产业发展。启动并推进文化产业“四千行动计划”，开办李可染画院和梅花河画家村，着手打造印象伊春主题文化演出项目。支持艺术团体围绕夜间旅游开展地域特色演艺活动。研究伊春地域特色菜系，建设特色餐饮一条街，推进“北红玛瑙北沉香旅游纪念品一条街”、“角园文化小巷”等文旅融合基地建设，下决心把夜场做起来。举办好2016第四届中国(东北亚)森林博览会，促进旅游与其它现代服务业共同繁荣。

大力发展木材精深加工业，突出资源整合和层次提升。木材加工业是我们传统优势最强、市场品牌影响较大、工业基础坚实的板块，必须努力克服原料供应不足、工艺落后、布局分散等短板，优化组合，重整旗鼓，重振雄风，打造中国实木家具之都。通过体制机制创新、工艺创新和营销模式创新，让这一传统优势产业焕发青春。支持光明、友春等品牌家居做大做强，发挥龙头带动作用，引导中小企业联合，实现品牌化。支持双丰家具园区建设，推进集群化发展。支持根雕木艺、玉石玛瑙产业做大做强，完善工艺品行业标准和规范，支持美江、侨艺、凯瑞林、永达、骊缘等木艺企业及红光玛瑙、昊林玛瑙、御达玉品等企业着力提高产品创意、雕刻水平，提高“中国木艺之乡”、“北红玛瑙之乡”和桃山玉文化影响力。

大力发展绿色矿业，突出盘活存量和做大增量。绿色矿业是我们资源替代能力最强、支撑能力最大的板块，必须正确处理好开发与保护的关系，努力克服产品层次低、产业链条短等短板，大力发展矿业经济。抓住省促进矿业经济发展的战略机遇，坚持向矿产资源开发加工要增量。支持鹿鸣钼矿精深加工项目建设，延伸产业链，提高附加值。鼓励西钢拓展融资渠道，加快战略重组。支持北方水泥研发新型建筑材料。持续优化地质勘查环境，跟踪推进省地质勘查三年专项项目、招拍挂探矿权项目落地。清理矿业权，吸引实力企业参与地质找矿。加快翠宏山铁多金属矿以及桃山玉、北红玛瑙矿规范有序开采和高锶矿泉水、地热等矿产资源开发利用。

大力开展项目园区建设与招商引资，突出项目落地和达产增效。全力推进和保障产业项目建设，促进其尽快落地、投产、达产。全年安排市级重点项目38个。加快铁力和翠峦园区基础设施建设，完善园区功能。铁力园区力争实现招商引资30亿元，晋级省级经开区。翠峦园区深入实施“三年攻坚战”和“180招商计划”，推动产业集群发展。加强与大公国际、中航工业、华电、丹东港集团、河南大白鹅集团等战略投资者的对接，力促项目落地转化，加快培育经济增长新动能。

(二)巩固提升生态建设水平。系统化推进生态文明先行示范区建设，全面增强生态保障力和林业生产力。

大力强化资源保护。以《党政领导干部生态环境损害责任追究办法》为准绳，严格落实生态保护主体责任。深入开展“森林资源保护专项行动”，严厉打击非法侵占、毁林开垦、盗伐盗挖盗猎及破坏湿地等行为，全面巩固停伐成果。抓好大平台航空灭火基地、市级森防物资储备库等项目建设，完善森防“四网四化”体系。加大对上政策争取，持续推进生态移民。正式启动伊春国家公园体制试点各项工作，探索生态保护、建设、管理新机制、新模式。

着力培育后备森林资源。充分考虑森林生产周期性，系统推进种苗繁育、造林更新、抚育经营，加快木材战略储备基地建设。支持国有苗圃企业化经营，推进苗木花卉产业发展。强化火烧迹地、林间空地和退耕还林地造林补植，加快推进红星50万亩生物质能源林建设。注重森林抚育向经营要效益，完成270万亩森林抚育经营任务。

严格推进节能减排。狠抓环境综合治理，对环境违法行为“零容忍”。继续推进重点工业企业脱硫、脱硝项目建设，加快淘汰黄标车，严格控制建成区新建20吨以下燃煤锅炉，确保空气质量优良天数保持在90%以上。

(三)加力深化改革开放。扭住关键，精准发力，不断把改革开放向纵深推进，激发林区后发优势。

深化国有林区改革。全面落实《国有林区改革指导意见》，突出抓好4个省市级试点，统筹推进13个政企合一区(局)管理体制改革，加快剥离企业办社会职能。创新国有森林资源管理体制，探索组建三级国有林业管理机构。积极探索生态补偿和碳汇交易机制，扩大汤旺河森林增汇减排、翠峦温室气体减排试点成果。鼓励支持森工企业及林业职工创办各类实体公司。加快创建新型经营主体和服务主体，推广“龙头企业+合作社(基地)+农(林)户”等模式。对全市130万亩农村集体土地进行确权登记。

着力激发市场主体活力。以提高经济运行效率为核心，着重支持非公经济健康发展。继续优化行政审批流程，加快推进“四证合一”等商事制度改革。全面推进 “质量伊春”和“诚信伊春”建设。认真执行结构性减税政策，进一步清理和规范涉企收费，切实帮助企业降成本、增效益。加强与大公国际、兴安汇、哈慈股份等集团对接，全面探索林业资源资本化运作路径。加大对实体经济的金融支持力度，新增小额贷款或融资性担保公司1-2家。推进社会资本通过PPP等方式进入公共事业领域。继续推动兴安新能源、忠芝等企业在新三板、创业板上市。

全面推进对外开放。积极融入“龙江丝路带”建设，力争嘉荫口岸早日复关，全力推动嘉荫跨境合作区和对俄互市贸易区规划建设。组建对俄经贸合作集团，扩大对俄土地和林业开发，全面拓展对俄经贸合作领域。加强与东北东部区域(12+2)城市交流合作，力争在交通建设、产业发展和生态保护等领域取得实质性突破。

(四)深入推进美丽伊春建设。坚持以人为本，突出规划引领，强化基础支撑，加强城市管理，不断提升城乡协调发展水平。

优化城镇布局。加快制定城镇集群和人口集聚规划，全力促进“四区融合”，率先加快伊春区与乌马河区融合步伐，着力推进新青、金山屯、南岔等节点城镇建设，增强中心区和节点城镇综合承载力、人口集聚力。继续加大林场所撤并整合力度，提高城镇化质量。

完善城乡基础设施。全力推进伊翠铁路改线、汤伊公路建设，开展市客运中心站建设前期工作。继续抓好保障房建设，支持国有及民营开发企业通过政府购买棚改服务、大户型改造等方式加速房地产“去库存”。推进中心城净水厂、燃气管道等设施建设，加大供热、供水、排水老旧管网改造力度。增加城镇绿量，提升绿化品位，中心林场、村屯道路硬化率、自来水入户率、污水处理率全部达标。加大水土保持生态建设力度，强化山洪预警，城市防洪以及重点水利工程完成年度建设任务。

提升精细化管理水平。加快推进“智慧伊春”建设，编制通信工程、海绵城市、地下管廊建设专项规划，完成地下综合管网普查。推行城市网格化管理，健全城市管理长效机制。深入开展市容环境综合整治，加强农贸市场、步行街等公共场所监管。推进物业管理市场化，支持长城物业等企业创新经营模式，增强服务能力。开展“创文”攻坚，推进“美丽伊春”建设，促进城乡公共资源均衡配置。

(五)扎实推动创新创业。突出创新在转型发展中的核心作用，以创新引领创业，以创业促进创新，激发“大众创业、万众创新”活力。

提升科技创新能力。继续推进“全省千户科技型企业三年行动计划”，全年新形成一定规模科技型企业5户以上。引导林科院、农研中心等科研院所与企业共建研发推广机制，与大连民族大学共建森林食品科学研究院，推进协同创新和资源共享。加大科技创新投入，实施重点科技项目25项。落实科技成果转化股权和分红激励政策，激发科研人员创新潜能。

加强创业平台建设。继续推进翠峦小微企业园、电商产业园、淘宝大学培训基地等创业孵化基地建设，支持利用闲置厂房、商业设施、清理腾退的非办公类用房，构建一批低成本、便利化、全要素众创空间。推进以“互联网+”为载体的创业模式培育。放大大学生创业“种子资金”等扶持政策效应，对符合条件的众创空间给予税费优惠和财政补贴。争创国家级小企业创业示范基地，扶持创业初期小企业540户。

强化人才引进和培养。加快推进招才引智工作，完善人才、智力、项目相结合的柔性引才机制。实施“人才兴安岭工程”，优化编制资源配置，加大急需紧缺人才引进力度。创新人才使用办法，打破单位和地域限制，为优秀人才搭建事业舞台。

(六)竭力增进民生福祉。坚持保基本、全覆盖、促公平，持续加大社会事业和民生投入，让全市人民在共建共享中有更多获得感。

抓好脱贫攻坚。坚持精准扶贫、精准脱贫，分类实施“五个一批”工程，确保19个省级贫困村两年内全部“摘帽”、4.1万农村贫困人口和林业困难职工全部脱贫。

提升社会保障能力。制定鼓励林业富余人员自主创业的具体办法，促进转岗职工和困难群体就业。落实稳岗补贴政策，发挥失业保险稳定就业的作用。新增就业 2万人，失业人员再就业1.4万人。继续抓好城乡低保标准和各类保险扩面，加强社会福利机构建设，着力解决低收入、残疾人等困难群体生活问题。

提升公共服务质量。全市87所义务教育学校全部达到国家评估验收标准，力争在全省率先实现均衡发展。优化高中教育布局，集中力量打造市一中等省、市重点高中。促进职业教育产教融合、校企合作。支持市老年大学向规范化、标准化方向迈进。继续推进医疗联合体建设。扩大医保即时结算范围，推进新农合异地就医即时结报。大力发展体育事业，广泛开展全民健身运动，承办国内外有影响力的10项赛事。实施“健康伊春”战略，提升市民健康水平。落实全面放开“二孩”国策，加强妇幼服务设施建设。全面推动公共文化服务体系建设及文化惠民工程。推进社区居家养老和日间照料机构建设，加快构建多元养老服务体系。

提升社会治理水平。健全公共安全体系，加强城镇防火、道路交通、食品药品等重点领域安全监管，推进企业安全生产标准化达标升级。完善社区综合服务，提高公益性社会服务供给能力和水平。全面推进重大决策社会稳定风险评估，运用法治思维和方式化解社会矛盾。不断提升“平安伊春”建设水平，促进社会和谐稳定。重视家庭建设，倡导良好家风，以家风带民风、促政风。

各位代表，尽管今年财政仍然处于困难期，但财政再难不能难百姓，财力再紧不能紧民生。我们要在大力紧缩财政支出、压缩“三公”经费的基础上，进一步集中有效财力，全力办好15件民生实事：

1、筹措资金，将厂办大集体职工经济补偿金和接续养老、医疗保险等政策落实到位。

2、启动政企合一区公务员登记，兑现公务员待遇。

3、实施医疗惠民工程，开展免费婚前检查，城镇职工基本医疗保险全部实现市级统筹。推进华夏急救培训联盟伊春中心建设，提高突发疾病救治能力。投资2亿元，在铁力市兴建一所三级医院，改善区域医疗条件，提升医疗水平。

4、将全市城市低保标准由月人均440元提高到500元;农村低保标准由年人均2700元提高到3500元。筹集资金7000万元，对突发性困难家庭给予应急性、过渡性救助。落实困难群体殡葬惠民政策，免收低保家庭的4项基本服务费用，让困者有其助，难者有其帮，逝者有尊严。

5、改造棚户区1.2万户(套)，改造农村泥草房1000户，对全市23个旧小区和112栋单体楼进行综合改造，将铁路棚户区纳入今年改造计划，对100万平方米旧楼房进行节能保温改造，让居住在棚户区的百姓搬进新房子，住上暖屋子。

6、投资3340万元对伊春火车站进行全面改造，对站内老旧工房全部拆除，对候车室功能进行完善提升，对站外广场、楼面统一绿化装修，从根本上改善百姓出行环境，擦亮伊春窗口，打造城市名片。

7、续建中心城水厂，整合改造中心城供热管网，华能热电实现全面联网供热。

8、完成市中小学生综合实践基地建设，全面提高学生综合素质。按照分级负担原则，免费为在职教师进行健康体检。

9、免费开放中央、黑龙江及伊春电视台相关频道，满足市民基本文化娱乐需求。

10、新建、改扩建300-500平方米达标和标准型社区服务中心3个，1000平方米示范型社区服务中心2个。

11、购置环保节能公交车60辆，持续提升公共交通服务水平。

12、调整环卫工人交通补贴政策，为全市一线环卫工人每人每年增加冬季作业补助200元。临街商店全面开通“暖心屋”工程，让环卫工人雨天工作可避雨，雪天作业可暖身，真正让普通劳动者享有关爱和尊重。

13、推进“健康伊春”建设，组织开展20项全民健身系列活动。改造升级伊春河右岸健康步道(彩虹桥至玉玺大桥段)及左岸自行车绿道(彩虹桥至沙滩浴场段)。省运会场馆及其它健身场所全部向市民开放。

14、建设设施齐全、管理规范、符合标准的松韵城农贸市场，解决农民卖菜难、新区居民买菜难的问题。

15、开工建设伊春儿童乐园，优化设施布局和安全管控，提高知识性、趣味性、娱乐性，让林都的孩子们真正拥有愉悦身心、开启心智的好去处。

(七)切实加强政府自身建设。全面完成今年各项目标任务，我们必须继续深入落实“三严三实”要求，推进政府工作向规范、依法、务实、高效方向转变。深入推进依法行政。继续推进简政放权，健全权力清单和责任清单制度。落实规范文明执法措施，完善政府法律顾问制度。强化权力监督制约，自觉接受人大依法监督、政协民主监督和社会公众监督，扩大政务公开范围。提高政府工作专业化水平。加强干部培训，强化专业思维、专业素养和专业方法，高度关注和适应新常态下增长动力转换的深刻变化，推动新技术、新产业、新业态、新商业模式带动经济发展。持续改进工作作风。巩固深化群众路线教育实践活动和“三严三实”专题教育成果，扎实开展“两学一做”活动，把纪律和规矩挺在前面，向省委省政府看齐，向市委看齐，始终保持担当尽责、开拓进取、务实高效的精神状态，为担当者担当，为尽责者尽责。坚持勤俭办一切事情，年内完成公车改革。完善行风热线、公开述职等沟通渠道，及时回应群众关切。持续优化发展环境，认真受理企业投诉，坚决惩处妨碍企业发展、插手工程建设以及慢作为、不作为、乱作为等行为。狠抓勤政廉政。强化绩效管理和行政监察，健全能定责、可追责的考核机制。强化制度规范，坚持用制度管权、管事、管人，加强政府采购、招投标管理，加大重点项目、重大资金和领导干部经济责任审计力度，严厉查处违法违纪行为，树立风清气正、阳光透明的政府形象。

各位代表、同志们，“十三五”的大幕已经开启，乐章奏响，时不我待。在新的起点推动伊春转型发展，是全市人民的殷切期待，更是政府的神圣职责。责任让我们义无反顾，使命砥砺我们奋力前行。让我们在市委的坚强领导下，以开拓创新的气魄、奋发有为的精神、敢于担当的勇气、团结务实的作风，凝心聚力、拼搏进取，攻坚克难，苦干实干，为把伊春全面建成小康社会而不懈努力!
